# Prognostic significance of gamma‐glutamyl transpeptidase to albumin ratio in patients with intrahepatic cholangiocarcinoma after hepatectomy

**DOI:** 10.1111/jcmm.17321

**Published:** 2022-04-28

**Authors:** Hui Li, Rongqiang Liu, Jiawang Li, Jiaxin Li, Hong Wu, Genshu Wang, Zhenhua Li, Dewei Li

**Affiliations:** ^1^ 605425 Department of Hepatobiliary Pancreatic Tumor Center Chongqing Key Laboratory of Translational Research for Cancer Metastasis and Individualized Treatment Chongqing University Cancer Hospital Chongqing China; ^2^ Department of Liver Surgery & Liver Transplantation West China Hospital Sichuan University Chengdu China; ^3^ Department of Hepatic Surgery and Liver Transplantation Center The Third Affiliated Hospital of Sun Yat‐sen University Guangzhou China; ^4^ Department of Head and Neck Surgery Chongqing Key Laboratory of Translational Research for Cancer Metastasis and Individualized Treatment Chongqing University Cancer Hospital Chongqing China

**Keywords:** gamma‐glutamyl transpeptidase to albumin ratio, hepatectomy, intrahepatic cholangiocarcinoma, prognosis

## Abstract

Inflammation has been reported to play an important role in tumour progression and prognosis. In this study, we evaluated the prognostic significance of γ‐glutamyl transpeptidase (GGT) to albumin ratio (GAR) in patients with intrahepatic cholangiocarcinoma (ICC) after hepatectomy. We retrospectively analysed 650 ICC patients underwent hepatectomy at three Chinese medical centres between January 2009 and September 2017. Patients were classified into derivation cohort (*n* = 509) and validation cohort (*n* = 141). Receiver operating characteristic (ROC) curve was used to determine the optimal cut‐off value for GAR. Survival curve and cox regression analysis were applied to assess the prognostic power of GAR. The prognostic accuracy of GAR was compared with other variables by ROC curve. The optimal cut‐off value for GAR was 1.3655. Preoperative high GAR was closely related to tumour number, lymph node invasion and GGT. The survival curve of derivation and validation cohorts showed that patients in the high GAR group had significantly shorter overall survival (OS) and disease‐free survival (DFS) than patients in the low GAR group. Multivariate analysis in the derivation cohort confirmed that GAR was an independent prognostic factor for survival outcomes. Moreover, the ROC curve revealed that GAR had better predictive accuracy than other variables. High GAR predicted poor OS and DFS in ICC patients after hepatectomy. GAR may be a novel, simple and effective prognostic marker for ICC patients.

## 1 INTRODUCTION

Cholangiocarcinoma (CCA) originating from bile duct epithelial cells is the second primary liver malignant tumour after hepatocellular carcinoma.^1^, ^2^ Intrahepatic cholangiocarcinoma is a type of cholangiocarcinoma occurring in secondary bile ducts and the branching epithelium.^3^ In recent years, epidemiological investigations have found that the incidence of ICC has a significant increasing trend globally.^4^ According to the survey, the incidence of cholangiocarcinoma was significantly higher in Asia than other regions.^5^ The main treatment for ICC was radical resection or liver transplantation. However, even with radical resection, the 5‐year survival rate was still <45%.^6^ Moreover, due to the strong aggressiveness and high degree of malignancy of ICC, many patients quickly had tumour recurrence after radical resection.^7^

The risk factors for ICC mainly include cirrhosis, viral hepatitis, chronic cholangitis and hepatolithiasis.^8^ These factors often lead to chronic inflammation of the liver and may induce ICC.^8^ Inflammation has been proved to play an important role in tumour progression.^9^ Inflammatory indicators have been confirmed to be closely related to the prognosis of different tumors.^10^ Currently, accumulating evidence has shown that inflammation‐based scores, such as systemic immune‐inflammation index (SII), platelet‐to‐lymphocyte ratio (PLR), neutrophil‐to‐lymphocyte ratio (NLR), lymphocyte‐to‐monocyte ratio (LMR), prognostic nutritional index (PNI), aspartate aminotransferase/platelet count ratio index (APRI), aspartate aminotransferase (AST) to neutrophil ratio index (ANRI), and AST to lymphocyte ratio index (ALRI) could be used as the prognostic markers for cholangiocarcinoma.^11^, ^12^, ^13^, ^14^, ^15^, ^16^, ^17^, ^18^ Gamma‐glutamyl transpeptidase (GGT) to albumin ratio (GAR) was a newly identified inflammation‐based score. Initially, GAR was pointed out to be useful for stratification of patients with chronic hepatitis B.^19^ Subsequent reports revealed that GAR could be used to predict the long‐term survival time of patients with hepatocellular carcinoma or pancreatic cancer after radical resection.^20^, ^21^ So far, no study has investigated the potential value of GAR in ICC patients after hepatectomy.

In this study, we firstly evaluated the clinical significance of GAR in ICC patients undergoing hepatectomy. In addition, we further compared the predictive power of GAR with other variables.

## 2 METHODS

This study complied with the ethical guidelines of the Declaration of Helsinki.^22^ We recruited a total of 650 ICC patients underwent hepatectomy at West China Hospital, the Third Affiliated Hospital of Sun Yat‐sen University and Chongqing University Cancer Hospital between January 2009 and September 2017. Inclusion and exclusion criteria were as follows: (1) ICC was confirmed by histopathology after hepatectomy; (2) no other tumours or metastasis; (3) no preoperative anti‐cancer treatments, chemotherapy or radiotherapy; (4) no history of decompensation due to the liver cirrhosis; (5) no gastrointestinal disorder with malnourishment; (6) no other underlying or chronic diseases; (7) no other history of inflammation or acute or chronic infection. Patients from West China Hospital were divided into derivation cohort, and patients from the Third Affiliated Hospital of Sun Yat‐sen University and Chongqing University Cancer Hospital were divided into validation cohort. Patients were followed every month at first 3 months post‐surgery, then every 3‐ to 6‐month for 2 years, and at 6‐month intervals thereafter. Imaging, most frequently ultrasonography and contrast‐enhanced CT, was performed at the time of 3 months after surgery and at least twice a year for the first 2 years. Besides, for those who determined not to go back to the hospital to re‐examination, a telephone follow‐up survey was performed. Only 8 (1.2%) patients were loss of follow‐up. The follow‐up period ended in January 2018. We collected clinical information of enrolled patients using electronic medical records. The main data included: age, sex, hepatitis virus infection, hepatolithiasis, tumour size, tumour number, differentiation, capsular invasion, perineural invasion, cirrhosis, microvascular invasion (MVI), tumour‐node‐metastasis stage (TNM), preoperative albumin and GGT. GAR was defined as preoperative gamma‐glutamyl transpeptidase divided by albumin. Overall survival (OS) was considered as the time from hepatectomy to death or last follow‐up for ICC patients. Disease‐free survival (DFS) was considered as the time from hepatectomy to recurrence or the last follow‐up for ICC patients. The Ethics Committee of West China Hospital, the Third Affiliated Hospital of Sun Yat‐Sen University and Chongqing University Cancer Hospital approved the study. All patients signed the informed consent form.

### 2.1 Statistical analysis

The optimal cut‐off value of GAR was determined by receiver operating characteristic (ROC) curve. We divided the derivation and validation cohorts into high and low GAR groups based on the optimal cut‐off value. The independent sample T‐test or the KruskaI Wallis test was used to analyse continuous variables. Fisher's exact test or the chi‐square test was used to explore categorical variable. The Kaplan–Meier curve was applied to describe survival data. Cox regression model was used for univariate and multivariate analysis to explore the valuable prognostic factors. The predictive ability of GAR was evaluated by the area under ROC curve. SPSS Software (Version 26.0; Chicago, IL, United States) and GraphPad Prism (Version 8.0, San Diego, CA, United States) were used for data analysis. *p* < 0.05 was considered statistically significant.

## 3 RESULTS

### 3.1 Correlation between GAR and clinicopathological characteristics

A total of 509 patients (249 male, 48.9%) from West China Hospital were included in the derivation cohort. 141 patients (72 male, 51%) from the Third Affiliated Hospital of Sun Yat‐sen University and Chongqing University Cancer Hospital were included in validation cohort (Table 1). The optimal cut‐off value for GAR was 1.3655. According to the optimal cut‐off value of GAR, patients in the derivation and validation cohort were divided into the GAR < 1.3655 group and the GAR > 1.3655 group. In the derivation cohort, tumour size (*p* = 0.002), tumour number (*p* = 0.032), node invasion (*p* < 0.001), perineural invasion (*p* = 0.021), GGT (*p* < 0.001) and OS were observed significantly different in the two groups. No differences were found in other indexes, such as Age, Gender, HBsAg, hepatolithiasis, differentiation, capsular invasion, MVI, cirrhosis, TNM stage and ALB. The clinicopathological information of patients in the validation cohort was shown in Table S1. Comparison of clinicopathological characteristics between derivation and validation cohort was displayed in Table S2.

**TABLE 1:** Correlation between GAR grade and clinicopathological characteristics in 509 ICC patients from derivation cohort

Variables	All patients (*n* = 509)	GAR grade		*p*‐value
		Low (*n* = 218)	High (*n* = 291)	
Age, ≤50/>50	135/374	59/159	76/215	0.840
Gender, male/female	249/260	105/113	144/147	0.833
HBsAg, +/−	149/360	67/151	82/209	0.598
Hepatolithiasis, +/−	72/437	30/188	42/249	0.842
Tumour size, <5/≥5	207/302	106/112	101/190	0.002
Tumour number, single/multiple	354/155	163/55	191/100	0.032
Differentiation, well/moderate‐poor	19/490	9/209	10/281	0.814
Capsular invasion, +/−	327/182	146/72	181/110	0.304
MVI, +/−	50/459	17/201	33/258	0.229
Node invasion, +/−	122/387	35/183	87/204	<0.001
Perineural invasion, +/−	73/436	22/196	51/240	0.021
Cirrhosis, +/−	140/369	58/160	82/209	0.764
TNM stage, I‐II/III	147/362	63/155	84/207	0.993
ALB	42.8 (40.0, 45.1)	43.6 (41.0, 45.4)	42.3 (39.2, 44.9)	0.116
GGT	69 (34, 152)	30 (22, 44)	133 (82, 224)	<0.001
Overall survival, months, mean (95% CI)	25.3 (23.5, 27.2)	29.6 (26.5, 32.7)	22.2 (19.9, 24.5)	<0.001

Abbreviations: ALB, albumin; CI, confidence interval; GAR, gamma‐glutamyltransferase to albumin ratio; GGT, gamma‐glutamyltransferase; ICC, intrahepatic cholangiocarcinoma; MVI, microvascular invasion; TNM, tumour‐node‐metastasis.

### 3.2 Prognostic analysis for GAR

In the derivation cohort, univariate analysis showed that hepatolithiasis (*p* = 0.038), tumour size (*p* = 0.039), tumour number (<0.001), MVI (*p* = 0.001), node invasion, (*p* < 0.001), perineural invasion, (*p* = 0.005), 8th TNM stage (*p* = 0.035), ALB (*p* = 0.009) and GAR (<0.001) were risk factors for adverse OS. However, multivariate analysis showed that tumour number (HR = 1.640, 95% CI: 1.279–2.103, *p* < 0.001), MVI (HR = 1.378, 95% CI: 1.103–1.722, *p* = 0.005), node invasion (HR = 1.991, 95% CI):1.515–2.617, *p* < 0.001) and GAR (HR = 1.655, 95% CI: 1.286–2.129, *p* < 0.001) were independent risk factors for poor OS. Similarly, tumour size (*p* < 0.001), tumour number (*p* < 0.001), MVI (*p* < 0.001), node invasion (*p* < 0.001), 8th TNM stage (*p* = 0.005) and GAR (*p* < 0.001) were closely related to poor DFS in univariate analysis. Multivariate analysis suggested that tumour number (HR = 1.582, 95% CI: 1.261–1.985, *p* < 0.001), MVI (HR = 1.608, 95% CI: 1.156–2.236, *p* = 0.005) and GAR (HR = 1.524, 95% CI: 1.219–1.906, *p* < 0.001) were independent risk factors for poor DFS. Detailed results were presented in Table 2.

**TABLE 2:** Cox regression analyses for DFS and OS of ICC patients in the derivation cohort

Variables	DFS			OS		
	Univariate *p*‐value	Multivariate *p*‐value	Multivariate HR (95% CI)	Univariate *p*‐value	Multivariate *p*‐value	Multivariate HR (95% CI)
Age, >50/≤50	0.950			0.359		
Gender, male/female	0.297			0.105	0.294	0.884 (0.701–1.113)
HBsAg, +/−	0.141	0.115	1.324 (0.938–1.869)	0.431		
Hepatolithiasis, +/−	0.877			0.038	0.179	1.227 (0.911–1.652)
Tumour size, ≥5/<5	<0.001	0.018	1.320 (1.049–1.660)	0.039	0.460	1.099 (0.856–1.411)
Tumour number, multiple/single	<0.001	<0.001	1.582 (1.261–1.985)	<0.001	<0.001	1.640 (1.279–2.103)
Differentiation, moderate‐poor/well	0.273			0.580		
Capsular invasion, +/−	0.110	0.766	0.936 (0.607–1.445)	0.535		
MVI, +/−	<0.001	0.005	1.608 (1.156–2.236)	0.001	0.005	1.378 (1.103–1.722)
Node invasion, +/−	<0.001	0.005	1.482 (1.129–1.946)	<0.001	<0.001	1.991 (1.515–2.617)
Perineural invasion, +/−	0.124	0.579	1.095 (0.795–1.509)	0.005	0.058	1.371 (0.990–1.899)
Cirrhosis, +/−	0.493			0.081	0.779	1.027 (0.852–1.238)
8^th^ TNM stage, III/I‐II	0.005	0.501	1.182 (0.726–1.924)	0.035	0.683	1.062 (0.796–1.418)
ALB	0.103			0.009		
GGT	0.767			0.168		
GAR grade	<0.001	<0.001	1.524 (1.219–1.906)	<0.001	<0.001	1.655 (1.286–2.129)

Abbreviations: ALB, albumin; CI, confidence interval; DFS, disease‐free survival; GAR, gamma‐glutamyltransferase to albumin ratio; GGT, gamma‐glutamyltransferase; ICC, intrahepatic cholangiocarcinoma; MVI, microvascular invasion; OS, overall survival; TNM, tumour‐node‐metastasis.

**FIGURE 1:**
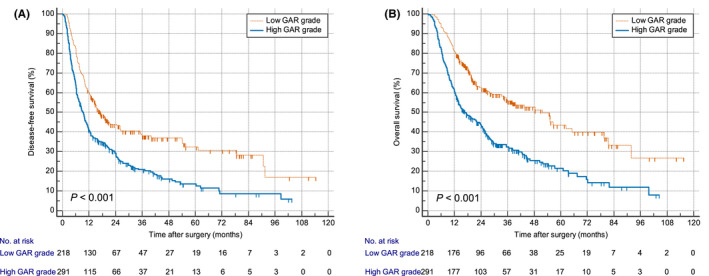
Kaplan–Meier curves for DFS and OS in low and high GAR group in the derivation cohort. (A) DFS, disease‐free survival. (B) OS, overall survival

**FIGURE 2:**
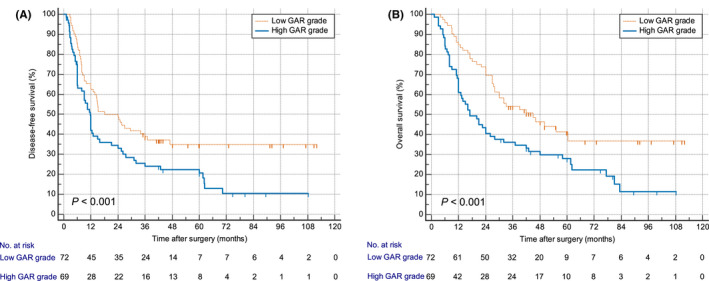
Kaplan–Meier curves for DFS and OS in low and high GAR group in the validation cohort. (A) DFS, disease‐free survival. (B) OS, overall survival

Furthermore, we used the Kaplan–Meier curve to analyse the relationship between GAR and survival outcome. In the derivation cohort, OS and DFS of patients in the high GAR group were significantly worse than those in the low GAR group (*p* < 0.001; Figure 1). The Kaplan–Meier curve results of the validation cohort were consistent with the derivation cohort (*p* < 0.001; Figure 2).

### 3.3 Comparison of the predictive accuracy of different markers

**FIGURE 3:**
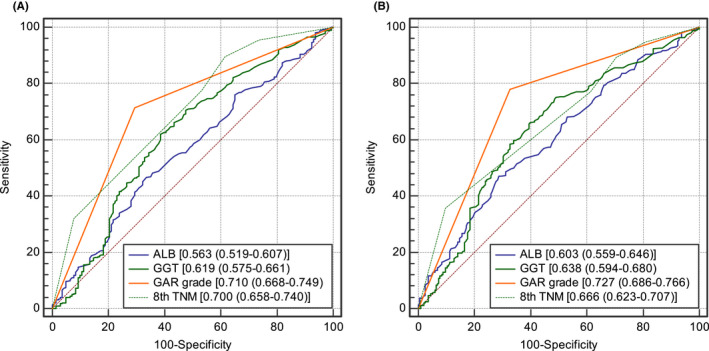
Comparison of the predictive accuracy of GAR and other variables in the derivation cohort. (A) DFS, disease‐free survival. (B) OS, overall survival

The predictive power of GAR was further discriminated using ROC curve. In the derivation cohort (Table S3), the area under ROC curve of GAR, ALB, GGT, 8th TNM stage, MVI, Node invasion and Tumour number for OS was 0.727, 0.603, 0.638, 0.666, 0.537, 0.606 and 0.586, respectively. The area under ROC curve of GAR, ALB, GGT and 8th TNM stage for DFS was 0.710, 0.563, 0.619, 0.700, 0.548, 0.597 and 0.616, respectively. Therefore, GAR had better predictive accuracy than other variables (Figure 3).

## 4 DISCUSSION

Since Balkwill firstly explored the association between inflammation and tumours, a large number of scholars have proved that inflammation plays an indispensable role in tumour progression.^23^, ^24^ GAR composed of gamma‐glutamyl transpeptidase and albumin is novel inflammatory marker. GGT as a ubiquitous epithelial enzyme was believed to be related to cardiovascular disease and chronic kidney disease.^25^, ^26^ Studies demonstrated that high GGT level was associated with the poor prognosis of patients with various tumours, including CCA.^27^, ^28^ Albumin is an important component in human plasma and a good indicator of liver dysfunction and malnutrition. The albumin level in the body obviously affected the survival time of cancer patient.^29^, ^30^ Given that both GGT and albumin influenced tumour progression, some scholars combined them to evaluate the prognostic ability of them in tumors.^31^, ^32^

The treatment and prognosis of ICC is still a thorny issue. A new diagnostic method called core biopsy has displayed potential value in judging the character of ICC.^33^ The prognosis of ICC remains poor despite the use of highly effective diagnostic tools and treatment methods. It is urgent to find effective prognostic markers for ICC patients. Effective markers can successfully help clinicians to identify ICC patients and timely implement individualized treatment.^34^, ^35^ In this retrospective study, we assessed prognostic value of GAR in ICC patients after hepatectomy for the first time. Through exploring the relationship between clinicopathological features and GAR, we found that preoperative high GAR was significantly correlated with tumour size, tumour number, mode invasion and perineural invasion, which suggested that high GAR may be associated with tumour proliferation, invasion and metastasis. Multivariate analysis revealed that high GAR was an independent risk factor for poor prognosis in ICC patients. We also observed that tumour number, MVI and node invasion also significantly affected the long‐term survival time of ICC patients, which was consistent with the previous studies.^35^, ^36^, ^37^ In addition, serum ALB level had an impact on OS in ICC patients.

γ‐glutamyl transpeptidase is believed to be mainly involved in the metabolism of glutathione, and protects cells from oxidative damage.^38^ High GGT levels can promote tumour cell proliferation, invasion and metastasis by increasing reactive oxygen species (ROS) and promoting oxidative stress.^39^ In addition, a previous study showed that GGT was involved in the degradation of the tumour suppressor PTEN.^40^ Studies also found that GGT could help tumour cells develop resistance to chemotherapeutic drug.^41^ At present, GGT has been recognized as a marker of oxidative stress and tumour risk in the body.^42^, ^43^ Serum GGT levels could be used as prognostic marker in a variety of different tumors.^27^ Albumin suppresses inflammatory responses by removing ROS and inhibiting oxidative stress.^44^ Studies have revealed that albumin can inhibit tumour cell proliferation as an anti‐tumour factor.^45^, ^46^ Decreased albumin levels indicated poor nutritional status, immune status and prognosis in cancer patient.^47^ It is reasonable to combine albumin with γ‐glutamyltransferase,which can reflect the patient's liver function, inflammatory response and immune status to evaluate the prognosis of liver cancer patients. Studies successively revealed that Albumin to gamma‐glutamyltransferase ratio(AGR) and gamma‐glutamyl transpeptidase to albumin ratio(GAR) could be as the prognostic indicators in ICC and HCC patients.^21^, ^32^ Combined with the above studies, we believed that GAR can effectively predict the prognosis of ICC patients.

Some flaws were still unavoidable in this study. Firstly, this study was a small sample retrospective study. Secondly, this was a retrospective study involving three centres. There were potential differences in surgical techniques and perioperative management between these centres. Thirdly, this study only evaluated the prognostic value of GAR in ICC patients after hepatectomy, and we did not assess the prognostic value of GAR in patients treated with TACE or liver transplantation. Finally, we did not compare GAR with some other blood markers, such as neutrophil‐to‐lymphocyte ratio (NLR) due to insufficient data.

In summary, our study suggested that high GAR predicted poor OS and DFS in ICC patients after hepatectomy. GAR may help to stratify ICC patients to identify high‐risk patients for individualized treatment. As an easily detectable blood inflammatory marker, GAR provided additional prognostic information for ICC patients and could be considered as a biomarker in the clinical management of ICC. More high‐quality randomized controlled studies were needed to test our findings and further examine the clinical value of GAR in ICC.

## CONFLICT OF INTEREST

The authors have declared no conflict of interest.

## AUTHOR CONTRIBUTIONS

**Hui Li:**Data curation (equal); Formal analysis (equal); Methodology (equal); Writing – original draft (equal). **Rongqiang Liu:** Data curation (equal); Formal analysis (equal); Methodology (equal); Writing – original draft (equal). **Jiawang Li:** Data curation (equal); Formal analysis (equal); Methodology (equal); Writing – original draft (equal). **Jiaxin Jiaxin Li:** Investigation (equal); Supervision (equal); Validation (equal). **Hong Wu:** Investigation (equal); Supervision (equal); Validation (equal). **Genshu Wang:** Investigation (equal); Supervision (equal); Validation (equal). **Dewei Li:** Funding acquisition (equal); Writing – review & editing (equal). **Zhenhua Li:** Data curation (equal); Writing – review & editing (equal).

## Supporting information

Table S1‐S3

## Data Availability

All data included in this study are available upon request by contact with the corresponding author.
