# Supplementary material for: Prognostic significance of gamma‐glutamyl transpeptidase to albumin ratio in patients with intrahepatic cholangiocarcinoma after hepatectomy
Source: J Cell Mol Med. 2022 Apr 28;26(11):3196–202. doi: 10.1111/jcmm.17321 (PMC9170822; doi:10.1111/jcmm.17321)
Supplement: Supplementary file 1 — Table S1‐S3 [file JCMM-26-3196-s001.docx]

Supplementary table1. Correlation between GAR grade and clinicopathological characteristics in external validation cohort.

| Variables | All patients | GAR grade | | | P value |
| --- | --- | --- | --- | --- | --- |
|  | (n=141) | | Low  (*n*=72) | High  (*n*=69) |  |
| Age, ≤50/>50 | 39/102 | | 22/50 | 17/52 | 0.457 |
| Gender, male/female | 72/69 | | 37/35 | 35/34 | 0.937 |
| HBsAg, +/- | 42/99 | | 24/48 | 18/51 | 0.364 |
| Hepatolithiasis, +/- | 22/119 | | 11/61 | 11/58 | 0.842 |
| Tumor size, <5/≥5 | 51/90 | | 34/38 | 17/52 | 0.008 |
| Tumor number, single/multiple | 101/40 | | 56/16 | 45/24 | 0.135 |
| Differentiation, well/moderate-poor | 7/134 | | 4/68 | 3/66 | 1.000 |
| Capsular invasion, +/- | 91/50 | | 44/28 | 47/22 | 0.481 |
| MVI, +/- | 11/130 | | 2/70 | 9/60 | 0.029 |
| Node invasion, +/- | 29/112 | | 9/63 | 20/49 | 0.021 |
| Perineural invasion, +/- | 15/126 | | 7/65 | 8/61 | 0.789 |
| Cirrhosis, +/- | 35/106 | | 17/55 | 18/51 | 0.846 |
| TNM stage, I-II/III | 40/101 | | 24/48 | 16/53 | 0.196 |
| ALB | 43.0 (41.0, 45.5) | | 43.5 (41.8, 45.8) | 42.7 (39.4, 45.3) | 0.062 |
| GGT | 58 (33, 73) | | 34 (26, 46) | 106 (76, 106) | <0.001 |
| Overall survival, months, mean (95% CI) | 34.5 (30.0, 38.9) | | 38.4 (32.4, 44.5) | 30.4 (23.8, 37.1) | <0.001 |

ICC, intrahepatic cholangiocarcinoma; MVI, microvascular invasion; TNM, tumor-node-metastasis; ALB, albumin; GGT, gamma-glutamyltransferase; GAR, gamma-glutamyltransferase to albumin ratio; CI, confidence interval.

Supplementary table2. Comparison of clinicopathological characteristics between derivation and validation cohort.

| Variables | All patients  (n=650) | Derivation  (n=509) | Validation  (n=141) | P value |
| --- | --- | --- | --- | --- |
| Age, ≤50/>50 | 174/476 | 135/374 | 39/102 | 0.830 |
| Gender, male/female | 321/329 | 249/260 | 72/69 | 0.704 |
| HBsAg, +/- | 191/459 | 149/360 | 42/99 | 0.917 |
| Hepatolithiasis, +/- | 94/556 | 72/437 | 22/119 | 0.061 |
| Tumor size, <5/≥5 | 258/392 | 207/302 | 51/90 | 0.381 |
| Tumor number, single/multiple | 455/195 | 354/155 | 101/40 | 0.679 |
| Differentiation, well/moderate-poor | 26/624 | 19/490 | 7/134 | 0.474 |
| Capsular invasion, +/- | 418/232 | 327/182 | 91/50 | 1.000 |
| MVI, +/- | 61/589 | 50/459 | 11/130 | 0.518 |
| Node invasion, +/- | 151/499 | 122/387 | 29/112 | 0.432 |
| Perineural invasion, +/- | 88/562 | 73/436 | 15/126 | 0.330 |
| Cirrhosis, +/- | 175/475 | 140/369 | 35/106 | 0.592 |
| TNM stage, I-II/III | 187/463 | 147/362 | 40/101 | 0.906 |
| ALB | 42.8 (40.3, 45.2 | 42.8 (40.0, 45.1) | 43.0 (41.0, 45.5) | 0.187 |
| GGT | 67 (34, 90) | 69 (34, 152) | 58 (33, 73) | 0.263 |

ICC, intrahepatic cholangiocarcinoma; MVI, microvascular invasion; TNM, tumor-node-metastasis; ALB, albumin; GGT, gamma-glutamyltransferase; GAR, gamma-glutamyltransferase to albumin ratio; CI, confidence interval.

Supplementary table 3. Comparison of the predictive accuracy of different markers in derivation cohort

.

|  | Accuracy | 95% CI | P-value |
| --- | --- | --- | --- |
| OS |  |  |  |
| -GAR grade | 0.727 | 0.686-0.766 | Ref. |
| -ALB | 0.603 | 0.559-0.646 | <0.001 |
| -GGT | 0.638 | 0.594-0.680 | <0.001 |
| -8^th^ TNM grade | 0.666 | 0.623-0.707 | 0.036 |
| -MVI | 0.537 | 0.493-0.581 | <0.001 |
| -Node invasion | 0.606 | 0.562-0.649 | <0.001 |
| -Tumor number | 0.586 | 0.542-0.630 | <0.001 |
| DFS |  |  |  |
| -GAR grade | 0.710 | 0.658-0.740 | Ref. |
| -ALB | 0.563 | 0.519-0.607 | <0.001 |
| -GGT | 0.619 | 0.575-0.661 | <0.001 |
| -8^th^ TNM grade | 0.700 | 0.658-0.740 | 0.752 |
| -MVI | 0.548 | 0.504-0.592 | <0.001 |
| -Node invasion | 0.597 | 0.553-0.640 | <0.001 |
| -Tumor number | 0.616 | 0.572-0.659 | <0.001 |

OS, overall survival; DFS, disease-free survival; GAR, gamma-glutamyltransferase to albumin ratio; ALB, albumin; GGT, gamma-glutamyltransferase; TNM, tumor-node-metastasis; MVI, microvascular invasion.
